# Cytotoxic Podophyllotoxin Type-Lignans from the Steam Bark of *Bursera fagaroides *var. *fagaroides*

**DOI:** 10.3390/molecules17089506

**Published:** 2012-08-09

**Authors:** Andrés M. Rojas-Sepúlveda, Mario Mendieta-Serrano, Mayra Y. Antúnez Mojica, Enrique Salas-Vidal, Silvia Marquina, María Luisa Villarreal, Ana María Puebla, Jorge I. Delgado, Laura Alvarez

**Affiliations:** 1 Centro de Investigaciones Químicas, Universidad Nacional Autónoma de México, Cuernavaca, Morelos 62209, Mexico; Email: amrojas@uaem.mx (A.M.R.-S.); antunezmayra@gmail.com (M.Y.A.-M.); smarquina21@hotmail.com (S.M.); 2 Departamento de Fisiología Molecular y Genética del Desarrollo, Instituto de Biotecnología, Universidad Nacional Autónoma de México, Cuernavaca, Morelos 62250, Mexico; Email: msma@ibt.unam.mx (M.M.-S.); esalas@ibt.unam.mx (E.S.-V.); 3 Centro de Investigación en Biotecnología, Universidad Autónoma del Estado de Morelos, Cuernavaca, Morelos 62209, Mexico; Email: luisav@uaem.mx; 4 Departamento de Farmacobiología, Centro Universitario de Ciencias Exactas e Ingeniería, Universidad de Guadalajara, Guadalajara, Jalisco 44430, Mexico; Email: ampuebla@cencar.udg.mx (A.M.P.); ivanovith@yahoo.com (J.I.D.)

**Keywords:** *Bursera fagaroides* var*. fagaroides*, lignans, podophyllotoxin, cytotoxic activity, antitumoral

## Abstract

The hydroalcoholic extract of the steam bark of *B. fagaroides* var. *fagaroides* displayed potent cytotoxic activity against four cancer cell lines, namely KB (ED_50_ = 9.6 × 10^−2^ μg/mL), PC-3 (ED_50_ = 2.5 × 10^−1^ μg/mL), MCF-7 (ED_50_ = 6.6 μg/mL), and HF-6 (ED_50_ = 7.1 × 10^−3^ μg/mL). This extract also showed anti-tumour activity when assayed on mice inoculated with L5178Y lymphoma cells. Bioactivity-directed isolation of this extract, afforded seven podophyllotoxin-type lignans identified as podophyllotoxin (**1**), β-peltatin-A-methylether (**2**), 5′-desmethoxy-β-peltatin-A-methylether (**3**), desmethoxy-yatein (**4**), desoxypodophyllotoxin (**5**), burseranin (**6**), and acetyl podophyllotoxin (**7**) by 1D and 2DNMR and FAB-MS analyses, and comparison with reported values. All the isolated compounds showed potent cytotoxic activity in the cell lines tested, especially compound **3**, which exhibited greater activity than camptothecin and podophyllotoxin against PC-3 (ED_50_ = 1.0 × 10^−5^ μg/mL), and KB (ED_50_ = 1.0 × 10^−5^ μg/mL). This is the first report of the isolation of podophyllotoxin and its acetate in a *Bursera* species.

## 1. Introduction

Podophyllotoxin (**1**) is one of the well-known bioactive naturally occurring aryltetralin lignans. This compound and its derivatives have great significance because of its biological activities, mainly as strong antineoplastic drugs and antiviral agents. Many semisynthetic derivatives of **1**, developed and tested for anticancer activity, have resulted in the commercial production of three glucosidic cyclic acetals of epipodophyllotoxin, that is, etoposide, teniposide, and etopophos. They are currently used in chemotherapy for various types of cancer, including small cell lung cancer, testicular carcinoma, lymphoma, and Kaposi’s sarcoma [1,2,3]. Some reviews on its distribution, sources, applications, synthesis and structure-activity relationship of podophyllotoxin have been published [1,4,5].

The genus *Bursera *(Burseraceae), which comprises approximately 100 species distributed from the southwestern United States to Peru, predominates in the tropical dry forests of México where about 85 species coexist and some 75 of them are endemic [6,7,8]. Several species produce an aromatic resin known as “copal”, which has been commonly burnt as incense in religious activities all over the country since ancient times [9,10]. The chemical profile of these plants includes flavonoids [11,12], triterpenes [13,14], sesquiterpenes [15,16], diterpenes [17], and lignans [18,19,20,21,22,23,24,25,26,27].

In Mexican traditional medicine a taxa complex of three *Bursera fagaroides* varieties (*B. fagaroides* var. *fagaroides*, *B. fagaroides* var. *elongata* and *B. fagaroides* var. *purpusii*) is described [28], which are reputed to have antitumor activity [29,30]. These are wild trees endemic to México and known as “aceitillo”, “copal” and “cuajiote amarillo”. Previous studies made on *B. fagaroides*, without specifying the variety studied, demonstrated that the chloroform extract showed antitumoral activity in the Walker carcinoma 256 tumor system WA16 [18], and the ethanol extract showed immunomodulator and antitumoral activities in the mouse lymphoma L5178Y cell line [31]. On the other hand, the ethanol extract from the bark of this plant affects the levels of polyamines, as well as the activity of the enzyme ornithine decarboxylase (ODC) *in vitro* and on the growth of *Entamoeba histolytica *[32]. It was also studied for its immobilization and agglutination effects on human and mouse spermatozoa [33]. Two lignans, β-peltatin-A-methylether (**2**) and 5′-desmethoxy-β-peltatin-A-methylether (**3**) from this plant were active against the WA16 tumor system [18]. Recently four podophyllotoxin related lignans, including deoxypodophyllotoxin, morelensin, yatein, and desmethoxy-yatein, were isolated from the cytotoxic ethanol extract of the dried exudates [19]. 

On the basis of the therapeutic potential of this plant as herbal drug, and in order to define its cytotoxic potential, we undertook a bioassay-guided isolation of the cytotoxic principles present in the hydroalcoholic extract obtained from the stem bark of one of the three varieties of this complex: *B. fagaroides* var. *fagaroides*.

In this paper we report on the antitumor and potent cytotoxic activities of the hydroalcoholic extract (HA) of the steam bark of *B. fagaroides* var. *fagaroides.* Purification of this extract by bioassay-guided chromatographic methods afforded seven podophyllotoxin-type lignans, which showed important cytotoxic activities against KB (nasopharyngeal), HF-6 (colon), MCF-7 (breast), and PC-3 (prostate) cancer cell lines with ED_50_ values comparable to those displayed by camptothecin, podophyllotoxin and etoposide used as positive controls.

## 2. Results and Discussion

**Figure 1:**
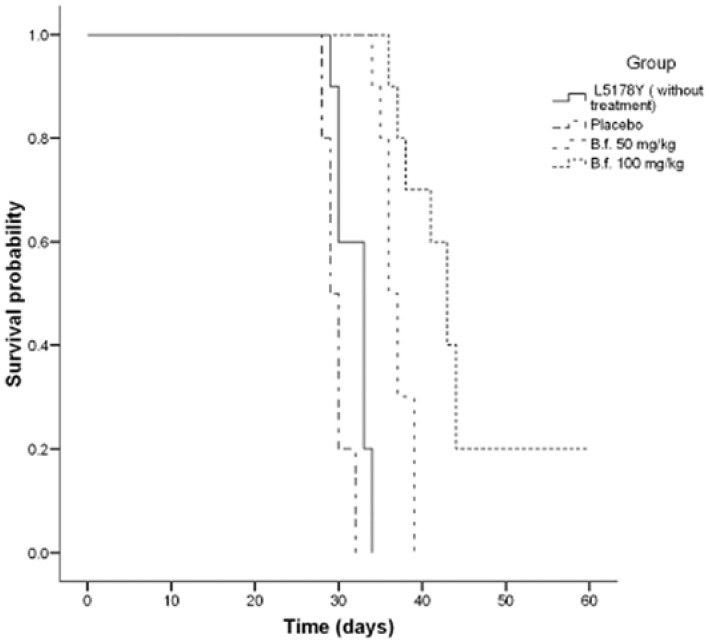
Antitumor activity of *Bursera fagaroides* var. *fagaroides *HA extract evaluated for the survival rate of mice with lymphoma L5178Y, comparing the control groups (without or placebo treatments) and versus treated groups with 50 and 100 mg/Kg/day/15 days (n = 10). All of them by Kaplan-Maier estimation of survival were different (*p *< 0.001).

The intraperitoneal administration of 50 and 100 mg/Kg of the hydroalcoholic extract of the bark of *B. fagaroides* var. *fagaroides* (HA), on mice inoculated with 2 × 10^4^ L5178Y lymphoma cells/mouse, showed an increase on the survival time (Figure 1). Mice with 2 × 10^4^ L5178Y cells usually die within 30 days without treatment. When treated with the dose of 100 mg/Kg of HA extract over 15 days, the survival was significantly prolonged (*p *< 0.001) compared with the control groups. Median survival time for the group without HA treatment was of 29 days, while for those that received the dose of 50 and 100 mg/Kg, this time increased to 35 and 38 days, respectively. The survival of the 100 mg/Kg group was 50% better than the 50 mg/Kg group (*p *< 0.05). The best response was observed with the 100 mg/Kg/day dose, where the survival of treated mice was significantly prolonged (*p *< 0.001) compared with the placebo and control groups. This dose cured 26% of the treated mice. Survival for more than 60 days without a tumor was considered to be a ‘cure’ [31]. On the other hand, this extract also significantly inhibited the proliferation of KB (ED_50_ = 9.6 × 10^−2^ µg/mL), PC-3 (ED_50_ = 2.5 × 10^−1^ µg/mL), HF-6 (ED_50_ = 7.1 × 10^−3^ µg/mL) and MCF-7 (ED_50_ = 6.6 µg/mL) tumor cell lines (Table 1).

**Table 1:** ED_50_ Values (μg/mL) of Extract, Fractions, and Active Compounds Isolated from *B. fagaroides* var. *fagaroides* against four human cancer cell lines.

Compound	KB	PC-3	MCF-7	HF-6
**HA**	9.6 × 10^−2^ ± 0.07	2.5 × 10^−1^ ± 0.03	6.6 ± 0.01	7.1 × 10^−3^ ± 0.1
**F-1**	6.0 × 10^−3^ ± 0.08	1.0 × 10^−5^ ± 0.006	8.8 × 10^−1^ ± 0.03	4.3 × 10^−3^ ± 0.04
**F-2**	1.3 × 10^−1^ ± 0.02	1.0 × 10^−5^ ± 0.004	8.2 × 10^−1^ ± 0.03	3.6 × 10^−2^ ± 0.02
**F-1-1**	3.94 × 10^−1^ ± 0.08	1.0 × 10^−5^ ± 0.1	8.1 ± 0.1	8.0 × 10^−5^ ± 0.03
**F-1-2**	3.5 × 10^−1^ ± 0.02	7.8 × 10^−4^ ± 0.06	1.3 ± 0.08	6.5 × 10^−3^ ± 0.01
**F-2-1**	1.9 × 10^−1^ ± 0.01	4.2 × 10^−3^ ± 0.02	>20	3.5 × 10^−2^ ± 0.02
**F-2-2**	3.2 ± 0.01	2.0 ± 0.1	>20	2.9 ± 0.05
**F-2-3**	1.0 × 10^−2^ ± 0.01	5.5 × 10^−3^ ± 0.01	2.5 × 10^−7^ ± 0.03	2.6 × 10^−3^ ± 0.001
**1**	1.91 × 10^−6^ ± 0.01	0.95 ± 0.005	1.04 × 10^−5^ ± 0.031	1.8 × 10^−4^ ± 0.01
**2**	0.189 ± 0.01	0.085 ± 0.005	0.798 ± 0.01	3.8 × 10^−2^ ± 0.01
**3**	1.0 × 10^−5^ ± 0.02	1.0 × 10^−5^ ± 0.004	1.02 × 10^−4^ ± 0.005	0.40 ± 0.01
**4**	0.4 ± 0.03	1.7 × 10^−3^ ± 0.01	0.4 ± 0.01	0.68 ± 0.01
**5**	1.5 ± 0.01	2.0 × 10^−3^ ± 0.003	1.25 ± 0.01	1.23 ± 0.01
**6**	2.89 ± 0.009	2.0 × 10^−3^ ± 0.005	3.68 ± 0.08	2.89 ± 0.006
**7**	1.03 ± 0.01	5.0 × 10^−3^ ± 0.005	>4	2.41 ± 0.004
Camptothecin	1.58 × 10^−3^ ± 0.01	0.96 ± 0.006	1.28 × 10^−4^ ± 0.01	5.5 × 10^−6^ ± 0.01
Podophyllotoxin	8.7 × 10^−5^ ± 0.003	0.85 ± 0.009	9.9 × 10^−5^ ± 0.005	7.6 × 10^−3^ ± 0.05
Etoposide	25 × 10^−3^ ± 0.002	5.6 × 10^−3^ ± 0.0005	0.54 ± 0.009	0.091 ± 0.02

**Figure 2:**
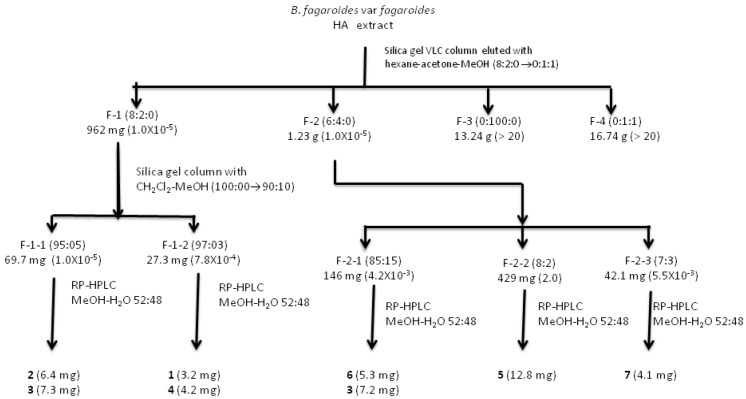
Fractionation tree diagram of the HA extract, monitored by the cytotoxic activity against PC-3 cell line in culture (ED_50_ values in μg/mL in square brackets).

Bioassay-guided isolation procedures, using the activity against KB, HF-6, MCF-7, and PC-3 cancer cell lines were carried out to define active components in this plant. Figure 2 shows the chromatographic fractionation of the HA extract monitored by the cytotoxic activity against PC-3 cells. Chromatographic fractionation of the HA extract afforded four fractions, two of which (F-1 and F-2), displayed potent cytotoxic activity against the four tested cell lines, principally against PC-3, both with ED_50_ values (1 × 10^−5^ μg/mL) greater than that displayed by the therapeutic drugs camptothecin (0.96 μg/mL), and etoposide (5.6 × 10^−3^ μg/mL) used as positive controls (Table 1).

**Figure 3:**
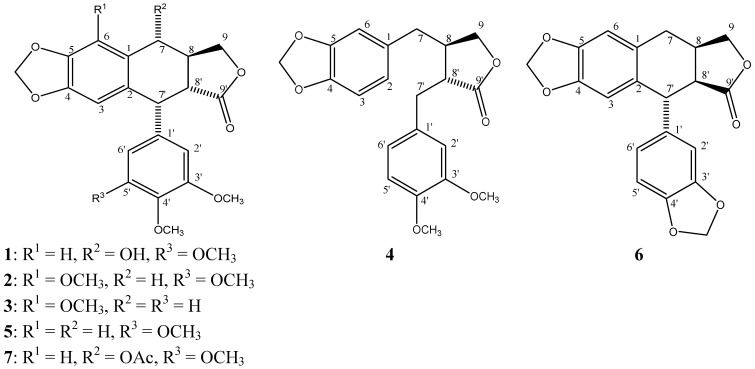
Structures of compounds **1**–**7**.

Chromatographic purification of F-1 afforded β-peltatin-A-methylether (**2**), 5′-desmethoxy-β-peltatin-A-methylether (**3**), desmethoxy-yatein (**4**), desoxypodophyllotoxin (**5**); while purification of F-2 afforded podophyllotoxin (**1**), burseranin, (**6**), and acetyl podophyllotoxin (**7**), which were identified by comparing their spectroscopic data with those previously described in the literature. The purity of isolated compounds was determined to be above of 95%, based on the peak areas of their HPLC chromatograms, as well as by their ^1^H-NMR spectra. The structures of these compounds are shown in Figure 3.

Podophyllotoxin (**1**) has traditionally been isolated from *Podophyllum peltatum* and *Podophyllum emodi*, but it has been found in around 20 genera such as *Diphylleia*, *Dysosma*, *Catharanthus*, *Polygala*, *Anthriscus*, *Linum*, *Hyptis*, *Teucrium*, *Nepeta*, *Thuja*, *Juniperus*, *Cassia*, *Haplophyllum*, *Commiphora*, and *Hernandia *[4]; β-peltatin-A-methylether (**2**) has been found in: *Juniperus phoenicea* [34], *B. permollis* [26], *B. fagaroides* [18], *B. simaruba *[35], *Anthriscus sylvestris *[36], *Libocedrus plumose* [37], and some *Linum* species and cultures [38,39,40,41], and its activity against A431, BC1, Col 2, HT, KB, LNCaP, Lu 1, Mel 2, U373, ZR-75-1 cancer cell lines [26], as well as its antitumor activity in the WA16 tumor system [18], have been described; 5′-desmethoxy-β-peltatin-A-methylether (**3**) has only been reported from *B. fagaroides* with activity against the WA16 tumor system [18]; desmethoxy-yatein (**4**) showed activity against P388 lymphocytic leukemia cell line [42], and has been isolated from *Hernandia ovigera* [43,44], *H. nymphaeifolia *[42], *Bursera schlechtendalii *[24], and *B. fagaroides* [19]; desoxypodophyllotoxin (**5**) is the most widespread aryltetralin lignan, and has been found in a great variety of plants belonging to various genera such as *Libocedrus *[37], *Linum *[41], *Bursera *[19], *Podophyllum *[45,46,47], *Anthriscus *[36], *Diphylleia *[47], *Dysosma *[48], *Hernandia *[42], among others, and its cytotoxic activity is well known [4]; burseranin (**6**) has been only described as a constituent of *B. graveolens*, and showed activity against HT1080 cell line [22]; acetyl podophyllotoxin (**7**) is a constituent of *Podophyllum*, and has been obtained from podophyllotoxin for structure-activity studies [1]. Until now, *Bursera* has been reported to contain only podophyllotoxin-related lignans, and this is the first report of the presence of podophyllotoxin (**1**) and its acetate **7** in a *Bursera* species.

Evaluation of the cytotoxic activity of the isolated compounds against the human cancer cell lines KB, PC-3, MCF-7, and HF-6 showed that, except for compounds **6** and **7**, all the isolated compounds displayed high activity (Table 1). The results showed that compound **3** exhibited the most potent cytotoxicity against PC-3 cells, with ED_50_ value of 1.0 × 10^−5^ µg/mL; whereas podophyllotoxin (**1**) displayed the most potent cytotoxiciy against KB and MCF-7 cells with ED_50_ values of 1.91 × 10^−6^, and 1.04 × 10^−5^ μg/mL, respectively. Remarkably, the cytotoxic activities of compounds **2**–**6** toward PC-3 cells were greater than those of podophyllotoxin (**1**), as well as the anticancer chemotherapy drugs camptothecin and etoposide (Table 1). On the other hand, acetyl podophyllotoxin (**7**), was the only compound that was not active towards MCF-7 cells, and together with burseranin (**6**), showed slight activity against KB, and HF-6 tumor cell lines, showing better activity against PC-3 (5.0 × 10^−3^, and 2.0 × 10^−3^ μg/mL, respectively), than etoposide. Compared with **1**, compound **2** displayed moderate activity against KB, PC-3 and MCF-7, with ED_50_ values ranging from 0.08 to 0.79 μg/mL, and showed the best activity against HF-6 cells with ED_50_ = 9.1 × 10^−2^. Compound **3**, displayed high activity against KB (ED_50_ = 1.0 × 10^−5^ μg/mL), PC-3 (ED_50_ = 1.0 × 10^−5^ μg/mL), and MCF-7 (ED_50_ = 1.02 × 10^−4^ μg/mL), and was moderately active against HF-6 (ED_50_ = 0.40 μg/mL). Compounds **4** and **5** displayed similar activities against the tested cell lines; the best activity was observed against PC-3 with ED_50_ values of 1.7 × 10^−3^, and 2.0 × 10^−3^ μg/mL respectively. It is interesting to point out that all of the lignans isolated from *B. fagaroides* var. *fagaroides* were active against PC-3 cells. 

The cytotoxic activity of podophyllotoxin (**1**), desoxypodophyllotoxin (**5**) and their congeners is well known [49,50,51,52]. Some structure-activity relationship studies, using several podophyllotoxin analogues, showed that the core structure of deoxypodophyllotoxin (**5**) is responsible for this cytotoxicity. The extra methoxy group on the 6-position in 5′-desmethoxy-β-peltatin-A-methylether (**3**) significantly changed the *in vitro *cytotoxicity when compared to desoxypodophyllotoxin (**5**). Compounds **4** and **6** which do not have the core structure of **5**, displayed less potent activity against KB, MCF-7 and HF-6, and were more selective against PC-3 cell line (Table 1). Results here obtained confirm the structure-activity relationships previously described, and provide new cytotoxic data for compounds **3**, **4**, **6** and **7** that complement the knowledge of this type of compounds.

Although the cytotoxicity of podophyllotoxin (**1**) and desoxypodophyllotoxin (**5**) is well known, the cytotoxicity of **3**, **4**, **6**, and **7** against the four cell lines tested, and of **2** against PC-3, MCF-7, and HF-6, is reported here for the first time.

## 3. Experimental

### 3.1. General

NMR spectra were acquired on a Varian Unity NMR spectrometer operating at 400 MHz for ^1^H and 100 MHz for ^13^C nuclei. Chemical shifts are listed in parts per million (ppm), referenced to CDCl_3_ and were made on the basis of ^1^H-^1^H gCOSY, ^1^H-^1^H TOCSY, NOESY, gHSQC and gHMBC spectral analysis as required. NMR experiments performed in CDCl_3_ are referenced to Me_4_Si (0 ppm). FABMS spectra in a matrix of *m*-nitrobenzyl alcohol or glycerol were recorded on a JEOL JMX-AX 505 HA mass spectrometer. All reagents and solvents used were analytical grade. Optical rotations were acquired with a Perkin-Elmer 241MC polarimeter (10 cm, 1 mL cell) at the sodium D line. High Performance Liquid Chromatography (HPLC) was performed using a Waters Delta Prep 4000 Module System equipped with a Waters 717 plus Autosampler and 996 Photodiode Array Detector (Waters Co., Milford, MA, USA), and a Xterra prep C18 column (5 μm, 7.8 × 100 mm) with MeOH-H_2_O (52:48) as the isocratic eluent system, UV detection at 215 nm and a flow rate of 1 mL/min.

### 3.2. Plant Material

The bark of *B. fagaroides* var. *fagaroides* (H.B.K.) Engl. was collected in the village of Capula between Zacapu and Quiroga, Michoacán, México. Its identification was made at the herbarium of the Instituto Mexicano del Seguro Social (registration number-12 051 IMSSM) and the Institute of Botany, University of Guadalajara (IBUG-140 748), México.

### 3.3. Extraction and Isolation

The stem bark from *B. fagaroides* var. *fagaroides* was dried under dark conditions at room temperature for 10 days. The dry material (1,420 g) was milled to obtain 2–5 mm particles and then extracted by successive percolation with *n*-hexane (3 L) and then with 70% aqueous methanol (MeOH, 3 L) at room temperature (three times). The obtained extracts were evaporated to dryness with a rotary evaporator under reduced pressure producing 6.14 g (0.43% yield) of *n*-hexane, and 33 g (6.51% yield) of hydroalcoholic dried extract, respectively. The hydroalcoholic extract was fractionated by percolation on a vacuum liquid chromatography column of silica gel (47 g) eluting with *n*-hexane-acetone-MeOH mixtures of increasing polarity to yield four fractions: F-1, 962 mg (8:2:0, 1.5 L), F-2, 1.23 g (6:4:0, 1.5 L), F-3, 13.24 g (0:100:0, 1.5 L), and F-4, 16.74 g (0:1:1, 3.5 L).

F-1 was chromatographed on silica gel (28.8 g) with a gradient mixture of CH_2_Cl_2_-MeOH (100:0→9:1) to give two active fractions: F-1-1 (69.7 mg), eluted with CH_2_Cl_2_-MeOH (95:5), and F-1-2 (27.3 mg), eluted with CH_2_Cl_2_-MeOH (97:3). An aliquot of 22.5 mg of F-1-1 was subjected to reverse-phase HPLC, to afford 6.4 mg of 5′-desmethoxy-β-peltatin-A methylether (**3**, t_R_ = 3.38 min), and 7.3 mg of desmethoxy-yatein (**4**, t_R_ = 4.21 min). The yields were based on peak areas of the HPLC chromatogram. An aliquot of 10.2 mg of F-1-2 was subjected to reverse-phase HPLC with the same conditions than F-1-1 to yield 3.2 mg of β-peltatin-A methylether (**2**, t_R_ = 13.95 min) and 4.2 mg of deoxypodophyllotoxin (**5**, t_R_ = 10.55 min). The yields were based on peak areas of the HPLC chromatogram.

F-2 was subjected to column chromatography packed with silica gel (50 g) and eluted with a gradient system of *n*-hexane-acetone (98:2→60:40) obtaining 110 fractions of 25 mL each. One of the active fractions (F-2-1, 0.146 g), eluted with 85:15 *n*-hexane-acetone, was subjected to column chromatography on silica gel (4.5 g), eluted with a gradient of *n*-hexane-acetone (95:5→8:2) to give 18.5 mg of a mixture of two compounds which were purified by HPLC with MeOH-H_2_O 52:48 as the isocratic eluent system, to afford 5.3 mg of acetyl podophyllotoxin (**7**, t_R_ = 8.08 min), and 7.2 mg of desmethoxy-yatein (**4**, t_R_ = 4.10 min). An aliquot of the second fraction, F-2-2 (429 mg), eluted with 8:2 *n*-hexane:acetone, was further purified by HPLC providing 12.8 mg of burseranin (**6**, t_R_ = 7.2 min). F-2-3, eluted with 7:3 *n*-hexane-acetone, was purified by HPLC with MeOH-H_2_O 52:48 as the isocratic eluent system to yield 4.1 mg of podophyllotoxin (**1**, t_R_ = 15.2 min). All the isolated compounds were identified using 1D and 2D NMR, optical rotation (OR), and HRMS analyses, and comparison with reported values. 

### 3.4. Spectral Data

*Podophyllotoxin* (**1**). White amorphous powder; purity = 98%; [α]^24^_D_ -133° (c 0.012, CHCl_3_); IR (KBr) γ_max_ 2932, 1778.9, 1727, 1241.0, 937.7 cm^−1^; ^1^H-NMR (CDCl_3_) δ 7.11 (1H, s, H-6), 6.50 (1H, s, H-3), 6.37 (2H, s, H-2′, H-6′), 5.97 (2H, dd, *J* = 6, 1.6 Hz, -O-CH_2_-O-), 4.61 (3H, m, H-7β, H-9α, H-7′), 4.07 (1H, dd, *J* = 10.8, 6 Hz, H-9β), 3.80 (3H, s, CH_3_O-4′), 3.75 (6H, s, CH_3_O-3′, CH_3_O-5′), 2.83 (2H, m, H-8, H-8′); ^13^C-NMR (CDCl_3_) δ 174.63 (C-9′), 152.83 (C-3′, C-5′), 148.03 (C-5), 147.91 (C-4), 137.49 (C-4′), 135.63 (C-1′), 133.37 (C-2), 131.39 (C-1), 110.01 (C-3), 108.66 (C-2′, C-6′), 106.5 (C-6), 101.66 (O-CH_2_-O), 71.54 (C-9), 60.96 (CH_3_O-4′), 56.6 (CH_3_O-3′, CH_3_O-5′), 45.52 (C-8), 45.53 (C-8′), 44.31 (C-7′), 40.99 (C-7); positive FAB-MS *m/z* 415 (20) [M + H]^+^, 413 (81), 391 (59), 355 (22), 327 (41), 467 (36), 239 (31), 221(90), 207 (100), 205 (57). These data match those in the literature [53].

*β-Peltatin A methyl ether *(**2**). White amorphous powder; purity = 96%; [α]^24^_D_ -113° (c 0.011, CHCl_3_); ^1^H-NMR (CDCl_3_) δ 6.8 (2H, s, H-2′, H-6′), 6.2 (1H, s, H-3), 5.8 (2H, s, O-CH_2_-O), 4.5 (1H, d, *J *= 4.4 Hz, H-7′), 4.4 (1H, dd, *J* = 8.8 Hz, H-9α), 3.9 (3H, s, CH_3_O-6), 3.8 (1H, dd, *J* = 10.4 Hz, H-9β), 3.7 (3H, s, CH_3_O-4′), 3.68 (6H, s, CH_3_O-3′, CH_3_O-5′), 3.1 (1H, dd, *J* = 4.8, 16 Hz, H 7β), 2.6 (1H, m, H-8′), 2.58 (1H, m, H-8), 2.4 (1H, dd, *J* = 10.4, 16 Hz, H-7α); ^13^C-NMR (CDCl_3_) δ 175.2 (C-9′), 152.9 (C-3′, C-5′), 148.6 (C-4), 141.1 (C-6), 136.4 (C-1′, C-4′), 135.1 (C-5), 132.0 (C-2), 121.2 (C-1), 109.0 (C-2′, C-6′), 104.7 (C-3), 101.2 (O-CH_2_-O), 72.6 (C-9), 59.6 (CH_3_O-6), 56.7 (CH_3_O-3′, CH_3_O-5′), 55.9 (CH_3_O-4′) 47.6 (C-8′), 44.1 (C-7′), 32.7 (C-8), 27.8 (C-7); positive FAB-MS *m/z* 428 [M + H]^+^ (66), 400 (13), 261 (32), 203 (15), 181 (24), 149 (51), 81 (100), 55 (98). These data match those in the literature [18,35].

*5′-Desmethoxy-β-peltatin A methylether* (**3**). White amorphous powder; purity = 99%; [α]^24^_D_ -140° (c 0.018, CHCl_3_); ^1^H-NMR (CDCl_3_) δ 6.9 (1H, d, *J* = 2.4 Hz, H-2′), 6.8 (1H, d, *J* = 8.4 Hz, H-5′), 6.4 (1H, dd, *J* = 8, 2 Hz, H-6′), 6.2 (1H, s, H-3), 5.9 (2H, d, *J* = 4.8 Hz, O-CH_2_-O), 4.5 (1H, d, *J* = 4.4 Hz, H-7′), 4.37 (1H, t, *J* = 6.8 Hz, H-9α), 4.0 (3H, s, CH_3_O-6), 3.85 (1H, dd, *J* = 10 Hz, H-9β), 3.8 (3H, s, CH_3_O-3′), 3.7 (3H, s, CH_3_O-4′), 3.1 (1H, dd, *J* = 4.8, 16 Hz, H-7α), 2.6 (1H, m, H-8′), 2.4 (1H, m, H-8), 2.3 (1H, dd, *J* = 10.4, 16 Hz, H-7β); ^13^C NMR (CDCl_3_) δ 175.4 (C-9′), 148.5 (C-5), 148.47 (C-4), 148.1 (C-6), 140.9 (C-4′), 134.9 (C-1′), 133.94 (C-3′), 132.2 (C-2), 129.0 (C-1), 122.8 (C-6′), 114.7 (C-2′), 110.5 (C-5′), 104.6 (C-3), 101.1 (O-CH_2_-O), 72.6 (C-9), 59.6 (CH_3_O-6), 56.2 (CH_3_O-3′), 56.0 (CH_3_O-4′), 47.5 (C-8), 43.5 (C-7′), 32.4 (C-8′), 29.9 (C-7); positive FAB-MS *m/z* 370 (18) [M + H]^+^, 313 (5), 279 (7), 257 (4), 178 (4), 149 (100), 95 (51), 57 (79). These data match those in the literature [18].

*5′-Desmethoxyyatein* (**4**). White amorphous powder; purity = 95%; [α]^24^_D_ -20° (c 0.018, CHCl_3_); ^1^H-NMR (CDCl_3_) δ 6.7 (1H, d, *J* = 7.6 Hz, H-6′), 6.67 (1H, d, *J* = 8 Hz, H-5′), 6.66 (1H, s, H-2′), 6.45 (1H, d, *J* = 7.6 Hz, H-3), 6.44 (1H, d, *J* = 8 Hz, H-2), 6.43 (1H, s, H-6), 5.9 (2H, d, *J* = 4 Hz, O-CH_2_-O), 4.1 (1H, dd, *J* = 7.2, 8.8 Hz, H-9β), 3.9 (3H, s, CH_3_O-3′), 3.84 (3H, s, CH_3_O-4′), 3.8 (1H, m, H-9α), 2.9 (1H, dd, *J* = 4.8 Hz, H-7′), 2.6 (1H, dd, *J* = 6.8 Hz, H-7β), 2.5 (2H, m, H-7α, H-8′), 2.48 (1H, m, H-8); ^13^C NMR (CDCl_3_) δ 178.8 (C-9′), 149.3 (C-3′), 148.2 (C-4′), 148.1 (C-5), 146.6 (C-4), 131.8 (C-1), 130.4 (C-1′), 121.8 (C-2), 121.6 (C-6′), 112. (C-2′), 109.0 (C-3), 111.4 (C-5′), 108.5 (C-6), 101.3 (CH_2_O_2_), 71.37 (C-9), 56.10 (CH_3_O-3′), 56.18 (CH_3_O-4′), 46.8 (C-8′), 41.3 (C-8), 38.6 (C-7), 34.9 (C-7′); positive FAB-MS *m/z* 370 (18) [M + H]^+^, 313 (5), 279 (7), 257 (4), 178 (4), 149 (100), 95 (51), 57 (79). These data match those in the literature [24].

*Desoxypodophyllotoxin *(**5**). White amorphous powder; purity = 99%; [α]^24^_D_ -104° (c 0.018, CHCl_3_); ^1^H-NMR (CDCl_3_) δ 7.1 (1H, s, H-6), 6.5 (1H, s, H-3), 6.37 (2H, s, H-2′, H-6′), 6.0 (2H, d, *J* = 1.2 Hz, O-CH_2_-O), 4.6 (1H, m, H-9α, H-7′), 4.0 (1H, dd, *J* = 10.8 Hz, H-9β), 3.8 (3H, s, CH_3_O-4′), 3.7 (6H, s, CH_3_O-3′, CH_3_O-5′), 2.8 (3H, m, H-7α, H-7β, H-8, H-8′); ^13^C-NMR (CDCl_3_) δ 174.6 (C-9′), 152.8 (C-3′, C-5′) 148.0 (C-5), 147.9 (C-4), 137.5 (C-4′), 135.6 (C-1′), 133.4 (C-2), 131.4 (C-1), 110.1 (C-3), 108.6 (C-6), 106.5 (C-6′, C-2′) 101.6 (O-CH_2_-O), 71.5 (C-9), 60.9 (CH_3_O-4′), 56.5 (CH_3_O-3′), 56.5 (CH_3_O-5′), 45.5 (C-8), 45.5 (C-8′), 44.3 (C-7′), 40.9 (C-7). These data match those in the literature [19].

*Burseranin *(**6**). White amorphous powder; purity = 96%; [α]^24^_D_ +34° (c 0.012, CHCl_3_); ^1^H-NMR (CDCl_3_) δ 6.7 (1H, d, *J* = 8.4 Hz, H-5′), 6.6 (1H, d, *J* = 8.0 Hz, H-6′), 6.59 (1H, s, H-2′), 6.3 (1H, s, H-3), 5.9 (2H, s, O-CH_2_-O-3′,4′), 5.8 (2H, s,), 4.4 (1H, dd, *J* = 9.2 Hz, H-9α), 4.3 (1H, d, *J *= 2.8 Hz, H-7′), 3.92 (3H, s, CH_3_O-6), 3.9 (1H, dd, *J* = 6.2 Hz, H-9β), 3.2 (1H, m, H-8′), 2.9 (1H, m, H-8), 2.7 (2H, m, H-7); ^13^C-NMR (CDCl_3_) δ 178.0 (C-9′), 147.9 (C-4), 147.5 (C-4′), 141.9 (C-3′), 140.5 (C-6), 135.2 (C-5), 131.8 (C-2), 121.0 (C-1′), 120.7 (C-1), 119.8 (C-6′), 108.3 (C-2′, C-5´), 104.1 (C-3), 100.72 (O-CH_2_-O-4,5), 100.7 (O-CH_2_-O-3′,4′), 73.0 (C-9), 59.5 (CH_3_O-6), 46.0 (C-8′), 44.7 (C-7′), 32.3 (C-8), 24.1 (C-7); positive FAB-MS *m/z* 382 [M + H]^+^ (0.4), 368 (0.4), 283 (0.6), 203 (0.5), 154 (40), 136 (35), 95 (75), 69 (91), 55 (100). These data match those in the literature [22].

*Acetyl podophyllotoxin *(**7**). White amorphous powder; purity = 99%; [α]^24^_D_ -146.0° (c 0.011, CHCl_3_); ^1^H-NMR (CDCl_3_) δ 6.7 (1H, s, H-6), 6.5 (1H, s, H-3), 6.3 (2H, s, H-2′, H-6′), 5.9 (2H, d, *J* = 1 Hz, O-CH_2_-O), 5.8 (1H, d, *J* = 8.4 Hz, H-7β), 4.5 (1H, d, *J* = 4 Hz, H-7′), 4.3 (1H, dd, *J* = 6.2, 9.0 Hz, H-9α), 4.1 (1H, dd, *J* = 9.4 Hz, H-9β), 3.75 (3H, s, CH_3_O-4′), 3.7 (6H, s, CH_3_O-3′, CH_3_O-5′), 2.8 (2H, m, H-8, H-8′), 2.1 (3H, s, CH_3_CO); ^13^C-NMR (CDCl_3_) δ 173.7 (C-9′), 171.4 (CH_3_CO), 152.6 (C-3′, C-5′), 147.6 (C-4), 148.1 (C-5), 137.1 (C-4′), 134.9 (C-1′), 132.4 (C-1), 128.3 (C-2), 109.8 (C-6), 108.1 (C-2′, C-6′), 107.1 (C-3), 101.7 (O-CH_2_-O), 73.7 (C-9), 71.5 (C-7), 60.9 (CH_3_O-4′), 56.28 (CH_3_O-3′, CH_3_O-5′), 45.7 (C-7′), 43.8 (C-8′), 38.8 (C-8), 21.3 (CH_3_CO); positive FAB-MS *m/z* 456 [M + H]^+^ (58), 397 (21), 313 (9), 229 (7), 185 (18), 154 (53), 136 (48), 95 (41), 77 (75), 55 (100), 41 (98). These data match those in the literature [19].

### 3.5. Cytotoxicity Assay

The *in vitro* cytotoxicity was measured by the sulphorhodamine B (SRB) (MP Biomedicals, LLC) protein staining assay [54,55] using KB (nasopharyngeal), HF-6 (colon), MCF7 (breast), and PC-3 (prostate) cancer cell lines. The cell cultures were maintained in RPMI-1640 medium supplemented with 10% fetal bovine serum, 5,000 units/mL penicillin, 5 mg/mL streptomycin, 7.5% NaHCO_3_, and cultured in a 96-well microtiter plate (10^4^ cells/mL, 190 μL/well) at 37 °C in a 5% CO_2_-air atmosphere (100% humidity). The cells at the log phase of growth were treated in triplicate (n = 3) with different concentrations of the test compounds (0.16, 0.8, 4 and 20 μg/mL), and incubated for 72 h. The cell concentration was determined by protein analysis. The optical density was measured at 590 nm with an ELISA-Reader (Molecular Devices, SPECTRA max plus 384). Results were expressed as the concentration that inhibits 50% of control growth after the incubation period (IC_50_). The values were estimated from a semi-log plot of the extract concentration (μg/mL) against the percentage of viable cells. Camptothecin, etoposide, and podophyllotoxin were included as positive standards.

### 3.6. Antitumor Activity

Male BALB/c mice (6–8 weeks old, 22–26 g) were provided by the Centro de Investigación Biomedica de Occidente (CIBO-IMSS). A lymphoma L5178Y cell line was used derived from a thymic lineage (haplotype H-2d) tumor induced in DBA/2 mouse by methyl-cholanthrene adopted to an ascetic form, and maintained by intraperitoneal (i.p.) transplantation of 10 × 10^6^ cells/mouse every 15 days in syngenic BALB/c mice [31]. For this study, all procedures involving animals were performed according to protocols approved by NOM-062-ZOO-1999. Animals were inoculated i.p. with 0.1 mL of suspension of fresh ascitic fluid, containing L5178Y lymphoma (2 × 10^4^) cells/mouse on day zero. Treatment with HA extract started 24 h after inoculation at doses of 50 or 100 mg/kg oral rout/day during 15 days, each group containing five mice and were observed during 60 days.

### 3.7. Statistical Analysis

The results were analyzed using one-way ANOVA followed by Kaplan-Meier estimation of survival and Cox’s regression through the statistical package SPSS V.15.

## 4. Conclusions

Bioassay-guided isolation of the hydroalcoholic extract obtained from the steam bark of *B. fagaroides* var. *fagaroides* identified a family of seven related lignans, among which podophyllotoxin (**1**) and acetyl podophyllotoxin (**7**) are described by the first time in *Bursera*. The presence of podophyllotoxin (**1**), together with six other related lignans in the cytotoxic extract of *B. fagaroides* var. *fagaroides *is noteworthy. In summary the cytotoxic and antitumor activities observed for *B. fagaroides* var. *fagaroides* are ascribable to the lignans present in this extract. Investigation of the podophyllotoxin-related lignans obtained from *B. fagaroides* var. *fagaroides* may lead to new cytostatic compounds, which could serve as the basis for new anti-tumor drugs. 
